# Rothman diagrams: the geometry of confounding and standardization

**DOI:** 10.1093/ije/dyae139

**Published:** 2024-11-14

**Authors:** Eben Kenah

**Affiliations:** Division of Biostatistics, College of Public Health, The Ohio State University, Columbus, OH, USA

**Keywords:** Confounding, L’Abbé, plots, standardization

## Abstract

We outline a geometric perspective on causal inference in cohort studies that can help epidemiologists understand the role of standardization in controlling for confounding. For simplicity, we focus on a binary exposure X, a binary outcome D, and a binary confounder C that is not causally affected by X. Rothman diagrams plot the risk of disease in the unexposed on the *x*-axis and the risk in the exposed on the *y*-axis. The crude risks define a point in the unit square, and the stratum-specific risks at each level of C define two other points in the unit square. Standardization produces points along the line segment connecting the stratum-specific points. When there is confounding by C, the crude point is off this line segment. The set of all possible crude points is a rectangle with corners at the stratum-specific points and sides parallel to the axes. When there are more than two strata, standardization produces points in the convex hull of the stratum-specific points, and there is confounding if the crude point is outside this convex hull. We illustrate these ideas using data from a study in Newcastle, United Kingdom, in which the causal effect of smoking on 20-year mortality was confounded by age.

Key Messages

- Rothman diagrams, where the risk of disease in the unexposed is plotted on the *x*-axis and the risk in the exposed is plotted on the *y*-axis, provide a geometric perspective on the control of confounding using standardization.
- When stratifying by a discrete covariate C, each stratum generates a point in the unit square. Standardized risks of disease under exposure and no exposure produce a point in the convex hull of these stratum-specific points.
- There is confounding by C if the crude association point is outside this convex hull. When stratifying by C is sufficient to control confounding, each point inside the convex hull has a causal interpretation for some distribution of C.
- The set of all possible crude association points is the circumscribing rectangle for the stratum-specific points. This can be used to find upper and lower bounds for measures of association under confounding by C.

## Rothman diagrams

The unit square is the set of all ordered pairs $(x,y)\in R^{2}$ where both x and y are in the interval [0,1]. To represent a point (x,y) in the unit square, we can plot x on a horizontal axis (the *x*-axis) and y on a vertical axis (the *y*-axis). In 1975, Rothman^1^ introduced a geometric perspective on causal inference where x represented the risk of disease in the unexposed or untreated and y represented the risk of disease in the exposed or treated. In 1987, L’Abbé *et al.*^2^ introduced a similar plot for meta-analysis, and these ‘L’Abbé plots’ have been used by Richardson *et al.*^3^ to discuss the estimation of risk differences and risk ratios. Because of historical precedence, we will call these pictures ‘Rothman diagrams’ when applied to causal inference.

To show how Rothman diagrams can help students understand the use of standardization to control confounding, we will use an example of confounding given by Appleton *et al.*^4^ that occurred in a cohort study of thyroid and heart disease among women in Newcastle, United Kingdom, in 1972–1974.^5^ Their smoking status was measured in the original study, and their 20-year survival status was measured in a follow-up study.^6^Table 1 shows the crude 2×2 table for smoking and 20-year mortality, and Table 2 shows the corresponding measures of association. All indicate higher 20-year mortality among nonsmokers (p=0.0024).

**Table 1:** Crude 2 × 2 table for smoking status and 20-year mortality from Appleton *et al.*^4^

	Dead	Alive	Total
Smoker	139	443	582
Nonsmoker	230	502	732
Total	369	945	1314

**Table 2:** Crude measures of association for smoking and 20-year mortality with likelihood ratio confidence limits and the *P*-value

Measure of association	Estimate	Likelihood ratio 95% confidence interval	LR *P*-value
Odds ratio	0.685	(0.535, 0.875)	0.0024
Risk ratio	0.760	(0.633, 0.908)	
Risk difference	−0.075	(−0.123, −0.027)	
Hazard ratio	0.724	(0.584, 0.892)	

Measures were estimated using binomial generalized linear models with the logit link for the odds ratio, the log link for the risk ratio, the identity link for the risk difference, and the complementary log-log link for the hazard ratio. The likelihood ratio *P*-value does not depend on the measure of association.

Table 3 shows 2×2 tables stratified by age at the time of the original survey. Older participants were less likely to smoke but more likely to die within 20 years than younger participants. Among those aged 18–64 years, the prevalence of smoking was 5331,072≈0.497 and the risk of death was 1621,072≈0.151. Among those aged ≥65 years, the prevalence of smoking was 49242≈0.202 and the risk of death was 207242≈0.855. The difference in survival between smokers and nonsmokers was due in part to different age distributions, so the causal effect of smoking on 20-year mortality was confounded by age.

**Table 3:** Age-stratified 2 × 2 tables for smoking and 20-year mortality adapted from Appleton *et al.*^4^

Participants aged 18–64 years				Participants aged ≥ 65 years			
	Dead	Alive	Total		Dead	Alive	Total
Smoker	97	436	533	Smoker	42	7	49
Nonsmoker	65	474	539	Nonsmoker	165	28	193
Total	162	910	1,072	Total	207	35	242

## Risks and points

Let X be a binary exposure or treatment (X=1 for the exposed and X=0 for the unexposed), D be a binary disease outcome (D=1 when disease occurs and D=0 otherwise), and C be a binary covariate that is not causally affected by X. The exposure, outcome, and covariate of individual i are $X_{i}$, $D_{i}$, and $C_{i}$, respectively. The covariate C could be defined by cross-classification of multiple covariates or by stratification of a propensity score,^7^^,^^8^ although problems can arise with residual confounding or data sparsity.

Let $D^{x}$ be the potential outcome when we intervene to set X=x.^9^ Under an intervention that sets $X_{i}=x$, the potential outcome of individual i is $D_{i}^{x}$. If $X_{i}=x$, then $D_{i}=D_{i}^{x}$ by consistency of potential outcomes,^10^ so we see the potential outcome associated with the actual or realized exposure of i.

In our example, X is smoking status at the time of the original survey, D is death within 20 years, and C is age. The potential outcome $D^{1}$ is death within 20 years as a smoker, and $D^{0}$ is death within 20 years as a nonsmoker. By consistency, $D=D^{1}$ for smokers and $D=D^{0}$ for nonsmokers. For each individual, only one of their potential outcomes is observed.

Probabilities are defined by proportions of the source population from which our study sample is drawn. For example, Pr(X=x,C=c) is the proportion of the population with X=x and C=c. Conditional probabilities are defined by proportions within subpopulations. For example, Pr(X=x∣C=c) is the proportion of the subpopulation with C=c that has X=x. Probabilities and conditional probabilities can be estimated by sampling individuals from the population, with uncertainty due to sampling variation summarized in terms of frequentist confidence intervals or Bayesian credible intervals. In our example, the study participants can be seen as a sample of size 1314 from the population of women in Newcastle between 1972 and 1974. For simplicity, we will assume no selection bias and ignore sampling variation whenever possible.

### Counterfactual risks and causal points

The counterfactual risk of disease when we intervene to set X=0 in the entire population is $\mathrm{Pr}(D^{0}=1)$, and the counterfactual risk of disease when we set X=1 is $\mathrm{Pr}(D^{1}=1)$. The point

(1) $$p_{\text{marg}}=(\mathrm{Pr}(D^{0}=1),\mathrm{Pr}(D^{1}=1))$$

on the Rothman diagram is the ‘marginal causal point’ for the population. It is causal because it is based on counterfactual risks of disease under intervention, and it is marginal because it ignores the covariate C.

In the subpopulation with C=c, the counterfactual risk of disease when we set X=x is:

(2) $$\mathrm{Pr}(D^{x}=1|C=c)=\frac{\mathrm{Pr}(D^{x}=1,C=c)}{\mathrm{Pr}(C=c)}$$

The point

(3) $$p_{c}=(\mathrm{Pr}\left(D^{0}=1|C=c\right),\mathrm{Pr}\left(D^{1}=1|C=c\right))$$

on the Rothman diagram is the ‘stratum-specific causal point’ for the subpopulation with C=c. There is a stratum-specific point for each level of C.

In practice, we are almost always missing the data needed to plot causal points on a Rothman diagram. To draw marginal or stratum-specific causal points, we need both potential outcomes $D_{i}^{1}$ and $D_{i}^{0}$ for each individual i in our sample. In our example, we would need to know whether smokers would have survived for 20 years as nonsmokers and whether nonsmokers would have survived for 20 years as smokers.

### Conditional risks and association points

**Figure 1:**
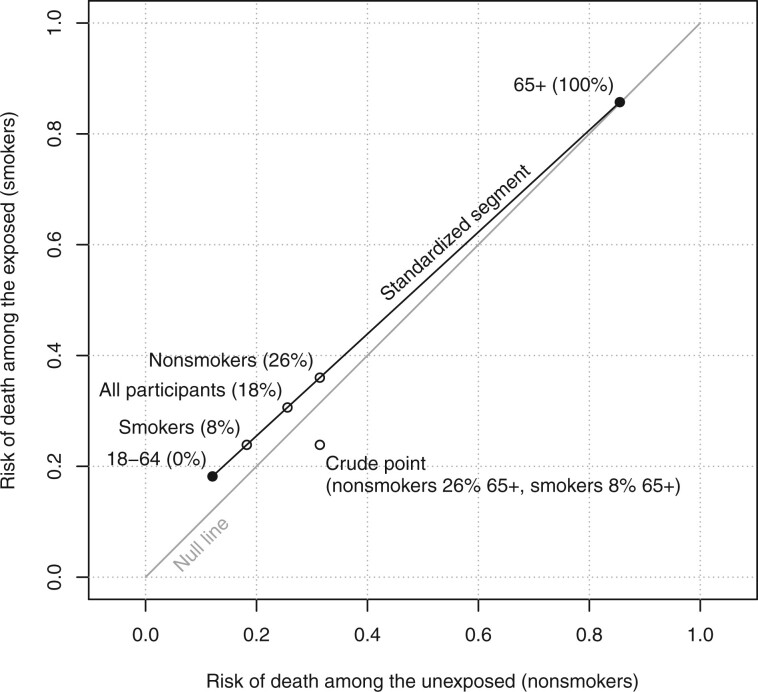
A Rothman diagram with the crude association point from Table 1 (open circle), the age-stratified association points from Table 3 (solid circles), the standardized segment (black line), and three standardized points (open circles). Each point is labelled with the proportion of its standard population in the 65+ year age group. The null line is the grey diagonal line

Unlike causal points, we can plot ‘association points’ based on observed data. The risk of disease among the unexposed is Pr(D=1∣X=0), and the risk of disease among the exposed is Pr(D=1∣X=1). The point

(4) $$p_{\text{crude}}^{*}=(\mathrm{Pr}(D=1|X=0),\mathrm{Pr}(D=1|X=1))$$

on the Rothman diagram is the ‘crude association point’ for the source population, which is approximated by the crude association point for the study sample. Figure 1 shows the crude point for the data from Table 1. Its *x*-coordinate is the risk of death among nonsmokers, which is 230702≈0.314. Its *y*-coordinate is the risk of death among smokers, which is 139582≈0.239. The ‘null line’ is the set of all points where the risks of death in the unexposed and the exposed are equal. It is a diagonal line from the point (0,0) to the point (1,1).

The conditional risk of disease given X=x and C=c is

(5) $$\mathrm{Pr}(D=1|X=x,C=c)=\frac{\mathrm{Pr}(D=1,X=x,C=c)}{\mathrm{Pr}(X=x,C=c)}.$$

The point

(6) $$p_{c}^{*}=(\mathrm{Pr}(D=1|X=0,C=c),\mathrm{Pr}(D=1|X=1,C=c))$$

is the ‘stratum-specific association point’ for the subpopulation with C=c. There is a stratum-specific association point for each value of C. Figure 1 shows these points for the age groups in Table 3. For both smokers and nonsmokers, the risks of death were much higher in the older age stratum, and the differences due to age were much larger than any differences due to smoking.

When there is no unmeasured confounding of the causal effect of X on D given C, exchangeability and consistency guarantee that

(7) $$\begin{array}{c} \mathrm{Pr}(D^{x}=1|C=c)=\mathrm{Pr}(D^{x}=1|X=x,C=c)\\ \begin{array}{c} =\mathrm{Pr}(D=1|X=x,C=c) \end{array} \end{array}$$

for x=0 and x=1.^10^^,^^11^ Thus, the stratum-specific association point $p_{c}^{*}$ equals the stratum-specific causal point $p_{c}$ in each stratum of C. In our example, the association points represent causal points (up to sampling variation) if stratifying into the 18–64 and 65+ year age groups removes all confounding of the association between smoking and 20-year survival. In reality, there is likely to be residual confounding by age or other covariates within these groups.

## Standardization and line segments

If we have two points $p_{0}=(x_{0},y_{0})$ and $p_{1}=(x_{1},y_{1})$ in $R^{2}$, we can use scalar multiplication and vector addition to produce another point in $R^{2}$:

(8) $$ap_{0}+bp_{1}=(ax_{0}+bx_{1},ay_{0}+by_{1}).$$

As time t goes from zero to one, the point

(9) $$p(t)=(1-t)p_{0}+tp_{1}$$

goes from $p_{0}$ to $p_{1}$ in a straight line. The line segment connecting $p_{0}$ and $p_{1}$ is the set

(10) $$\{(x,y)\in R^{2}:(x,y)=(1-t)p_{0}+tp_{1}\;\text{for}\;\text{some}\;t\in [0,1]\},$$

which is the set of all points that we encounter while going from $p_{0}$ to $p_{1}$ in a straight line.

For a binary covariate C, the marginal causal point must be on the line segment connecting the stratum-specific points. Because C is not causally affected by X, both Pr(C=0) and Pr(C=1) remain the same no matter how we intervene to set X. For any possible value x,

(11) $$\begin{array}{c} \mathrm{Pr}(D^{x}=1)=\mathrm{Pr}(D^{x}=1|C=0)\mathrm{Pr}(C=0)\\ +\mathrm{Pr}(D^{x}=1|C=1)\mathrm{Pr}(C=1) \end{array}$$

by the law of total probability. If $p_{0}$ and $p_{1}$ are the stratum-specific causal points for C=0 and C=1, then

(12) $$p_{\text{marg}}=p_{0}\mathrm{Pr}(C=0)+p_{1}\mathrm{Pr}(C=1).$$

Because Pr(C=0)+Pr(C=1)=1 and probabilities are nonnegative, the marginal causal point is on the line segment connecting $p_{0}$ and $p_{1}$.

With association points, line segments correspond to standardized risks of disease.^1^ The standardized risk of disease given X=x is the marginal risk of disease in a hypothetical ‘standard population’ where the prevalence of C=1 equals ${\text{Pr}}_{\text{std}}(C=1)$. The standardized risk of disease under X=x is

(13) $$\begin{array}{c} {\text{Pr}}_{\text{std}}(D=1|X=x)=\mathrm{Pr}(D=1|X=x,C=0){\text{Pr}}_{\text{std}}(C=0)\\ +\mathrm{Pr}(D=1X=x,C=1){\text{Pr}}_{\text{std}}(C=1). \end{array}$$

Standardization allows us to calculate marginal risks of disease using the same distribution of C for both exposure groups, which removes confounding by C. The corresponding ‘standardized association point’

(14) $$p_{\text{std}}^{*}=p_{0}^{*}{\text{Pr}}_{\text{std}}(C=0)+p_{1}^{*}{\text{Pr}}_{\text{std}}(C=1)$$

is on the line segment connecting the stratum-specific association points $p_{0}^{*}$ and $p_{1}^{*}$. Each point along this ‘standardized segment’ is a standardized association point for some standard population.

If there is no unmeasured confounding of the causal effect of X on D within strata of C and we standardize to the distribution of C in the population, then

(15) $$\begin{array}{c} p_{\text{std}}^{*}=p_{0}^{*}{\text{Pr}}_{\text{std}}(C=0)+p_{1}^{*}{\text{Pr}}_{\text{std}}(C=1)\\ \begin{array}{c} =p_{0}\mathrm{Pr}(C=0)+p_{1}\mathrm{Pr}(C=1)=p_{\text{marg}}, \end{array} \end{array}$$

so the standardized point equals the marginal causal point (up to sampling variation). We can also standardize to other distributions of C. Table 4 shows the three most common standard populations in causal inference with the interpretation of the standardized risks in terms of a hypothetical experiment. In a randomized trial, all three populations are the same up to sampling variation.

**Table 4:** Distribution of *C* for common standard populations and causal interpretation of each in terms of an experiment that compares the risks of disease in same population under two different exposures

Standard population	Pr_std_(*C* = 1)	Hypothetical experiment
Study sample	Pr(*C* = 1)	Study sample under *X* = 1
		versus themselves under *X* = 0
Exposed or treated	Pr(*C* = 1 | *X* = 1)	Exposed versus
		themselves under *X* = 0
Unexposed or untreated	Pr(*C* = 1 | *X* = 0)	Unexposed under *X* = 1
		versus themselves

Supplementary Material, Section S1, available as Supplementary data at *IJE* online shows that, in the limit of a large sample, the crude association point $p_{\text{crude}}^{*}$ is on the standardized segment connecting $p_{0}^{*}$ and $p_{1}^{*}$ if and only if the causal effect of X on D is unconfounded by C (or $p_{0}^{*}$ and $p_{1}^{*}$ share an *x*- or *y*-coordinate). In practice, the crude point will be slightly off the standardized segment due to sampling variation even when there is no confounding.

In our sample of 1314 women, there are 1072 women (81.6%) aged 18–64 years and 242 women (18.4%) aged 65+ years at the time of the original survey. Using this age distribution, the standardized risk of death among smokers is

(16) $$\left(\frac{1072}{1314}\times \frac{97}{533}\right)+\left(\frac{242}{1314}\times \frac{42}{49}\right)\approx 0.306$$

and the standardized risk of death among nonsmokers is

(17) $$\left(\frac{1072}{1314}\times \frac{65}{539}\right)+\left(\frac{242}{1314}\times \frac{165}{193}\right)\approx 0.256.$$

This standardized point is plotted in Figure 1. If stratifying by age has controlled confounding, the *x*-coordinate of this point is the estimated counterfactual risk of death in the study sample if we intervened to make all participants nonsmokers, and its *y*-coordinate is the estimated counterfactual risk of death if we intervened (unethically) to make all participants smokers.

Among the 582 smokers in the study sample, there are 533 women (91.6%) aged 18–64 years and 49 women (8.4%) aged 65+ years. Using this age distribution, the standardized risk of death among smokers is

(18) $$\left(\frac{533}{582}\times \frac{97}{533}\right)+\left(\frac{49}{582}\times \frac{42}{49}\right)=\frac{97+42}{582}\approx 0.239,$$

which is the marginal risk of death among smokers in the sample. The corresponding standardized risk of death among nonsmokers is

(19) $$\left(\frac{533}{582}\times \frac{65}{539}\right)+\left(\frac{49}{582}\times \frac{165}{193}\right)\approx 0.182.$$

This point is plotted in Figure 1. It has the same *y*-coordinate as the crude point. If stratifying by age has controlled confounding, then its *x*-coordinate is the estimated counterfactual risk of death among smokers if we intervened to make them nonsmokers.

Among the 732 nonsmokers in the study sample, there are 539 women (73.6%) aged 18–64 years and 193 women (26.4%) aged ≥65 years. Using this age distribution, the standardized risk of death among smokers is

(20) $$\left(\frac{539}{732}\times \frac{97}{533}\right)+\left(\frac{193}{732}\times \frac{42}{49}\right)\approx 0.360.$$

The corresponding standardized risk of death among nonsmokers is

(21) $$\left(\frac{539}{732}\times \frac{65}{539}\right)+\left(\frac{193}{732}\times \frac{165}{193}\right)=\frac{65+165}{732}\approx 0.314,$$

which is the marginal risk of death among nonsmokers in the sample. This point is plotted in Figure 1. It has the same *x*-coordinate as the crude point. If stratifying by age has controlled confounding, then its *y*-coordinate is the estimated counterfactual risk of death among nonsmokers if we intervened to make them smokers.

## Confounding and rectangles

The set in Equation (10) is a line segment connecting $p_{0}$ and $p_{1}$ because we use the same t for both axes. If we allow different values of t on each axis, we get the set

(22) $$\begin{array}{c} \{(x,y)\in R^{2}:x=(1-t_{x})x_{0}+t_{x}x_{1}\;\text{and}\;y=(1-t_{y})y_{0}+t_{y}y_{1}\\ \begin{array}{c} \text{for}\;\text{some}\;t_{x},t_{y}\in [0,1]\}, \end{array} \end{array}$$

which is a rectangle with sides parallel to the axes and corners at $p_{0}$ and $p_{1}$. It is called the ‘circumscribing rectangle’ for $p_{0}$ and $p_{1}$ because it is the smallest rectangle with sides parallel to the axes that contains both points. The line segment in Equation (10) is a diagonal of this rectangle. If $p_{0}$ and $p_{1}$ happen to share an *x*-coordinate or *y*-coordinate, this rectangle collapses into the line segment connecting them.

**Figure 2:**
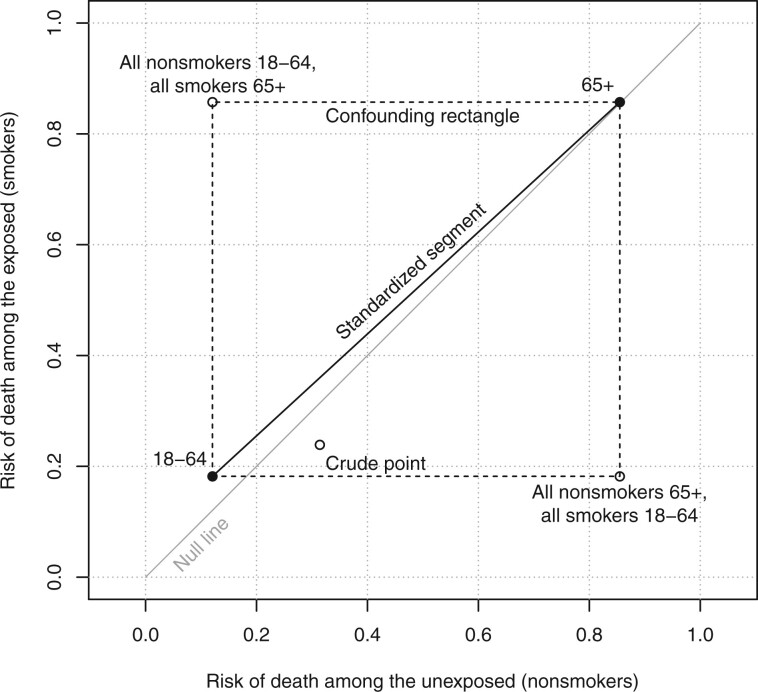
A Rothman diagram with the standardized segment (solid) and the confounding rectangle (dashed). The stratum-specific points define two corners of the rectangle. The other two corners represent the most extreme confounding possible given the stratum-specific risks of death in the exposed and unexposed

The crude association point $p_{\text{crude}}^{*}$ has the *x*-coordinate

(23) $$p_{0}^{*}\mathrm{Pr}(C=0|X=0)+p_{1}^{*}\mathrm{Pr}(C=1|X=0),$$

which corresponds to $t_{x}=\mathrm{Pr}(C=1|X=1)$ in Equation (22). It has the *y*-coordinate

(24) $$p_{0}^{*}\mathrm{Pr}(C=1|X=0)+p_{1}^{*}\mathrm{Pr}(C=1|X=1),$$

which corresponds to $t_{y}=\mathrm{Pr}(C=1|X=1)$ in Equation (22). Therefore, the crude point must be in the circumscribing rectangle for $p_{0}^{*}$ and $p_{1}^{*}$, either in its interior or on its perimeter.^1^ This is the ‘confounding rectangle’, and the standardized segment is one of its diagonals. Figure 2 shows a Rothman diagram with the confounding rectangle for the data in Table 3. The confounding rectangle always contains the crude point.

If there is confounding by C, then the distribution of C is different in the unexposed and the exposed so $t_{x}\neq t_{y}$. It follows that $p_{\text{crude}}^{*}$ is off the standardized segment (unless $p_{0}^{*}$ and $p_{1}^{*}$ happen to share an *x*- or *y*-coordinate). In Figure 2, the crude association point is off the standardized segment because of confounding by age.

Supplementary Material, Section S1, available as Supplementary data at *IJE* online shows that, in the limit of a large sample, the crude association point is off the standardized segment if and only if the causal effect of X on D is confounded by C (and $p_{0}^{*}$ and $p_{1}^{*}$ do not share an *x*- or *y*-coordinate). In practice, we can conclude that there is confounding if the distance between the crude point and the corresponding standardized point is sufficiently large compared with the distribution of distances that would be produced by sampling error alone.

The confounding rectangle can be used to find upper and lower bounds for any given measure of association under confounding by C.^1^ In Figure 2, the standardized risk difference must be between 0.002 (at the top right corner) and 0.06 (at the bottom left corner), but crude point has a risk difference of −0.075 (see Table 2). Given the same stratum-specific risks in smokers and nonsmokers, confounding by age group could produce a risk difference as low as −0.67 (in the bottom right corner) or as high as 0.74 (in the top left corner). Similar calculations can be done for other measures of association, including risk ratios, odds ratios, and hazard ratios.

## More than two strata

The generalization of the line segment in Equation (10) for k≥1 points $p_{1},\ldots ,p_{k}$ is the convex hull

(25) $$\begin{array}{c} \{(x,y)\in R^{2}:(x,y)=w_{1}p_{1}+\cdots +w_{k}p_{k}\;\text{for}\;w_{1},\ldots w_{k}\geq 0\\ \begin{array}{c} \text{such}\;\text{that}\;w_{1}+\cdots +w_{k}=1\}. \end{array} \end{array}$$

A sum of the points $p_{1},\ldots ,p_{k}$ where the weights are nonnegative and add up to one is called a ‘convex combination’ of the points. The ‘convex hull’ is the set of all convex combinations of $p_{1},\ldots ,p_{k}$. It is convex because it contains the line segment joining any two of its points. Intuitively, it is the shape you would get if you stretched a rubber band around all of the points. If k=2, the convex hull is the line segment connecting $p_{1}$ and $p_{2}$. If k=1, it is a single point.

Suppose that C has k≥1 possible values $c_{1},\ldots ,c_{k}$. Given k stratum-specific association points $p_{1}^{*},\ldots p_{k}^{*}$, any standardized point is a convex combination of these points, so it must be contained in the convex hull—either in its interior or on its perimeter. For k=2 strata, the convex hull is the standardized segment. For k>2 strata, we will call the convex hull of the stratum-specific points the ‘standardized hull’.

For any k≥2, we get a confounding rectangle (unless all of the points happen to share an *x*- or *y*-coordinate). Using different sets of weights for the exposed and unexposed, we can get any point in the circumscribing rectangle for $p_{1}^{*},\ldots ,p_{k}^{*}$. This rectangle always contains the entire standardized hull. Unlike the k=2 case, the confounding rectangle for k>2 strata does not necessarily have stratum-specific points at any of its corners.

When the causal effect of X on D is unconfounded by C, the crude point must be in the standardized hull. Each point in the standardized hull is a marginal causal point for some standard population. For k>2 strata, it is possible to get a crude point inside the standardized hull even when there is confounding, but the resulting risks in the exposed and unexposed are causal for some standard population. Supplementary Material, Section S1, available as Supplementary data at *IJE* online shows that, in the limit of a large sample, there must be confounding if the crude point is outside the standardized hull. Due to sampling variation, this implication is only approximate in practice.

**Figure 3:**
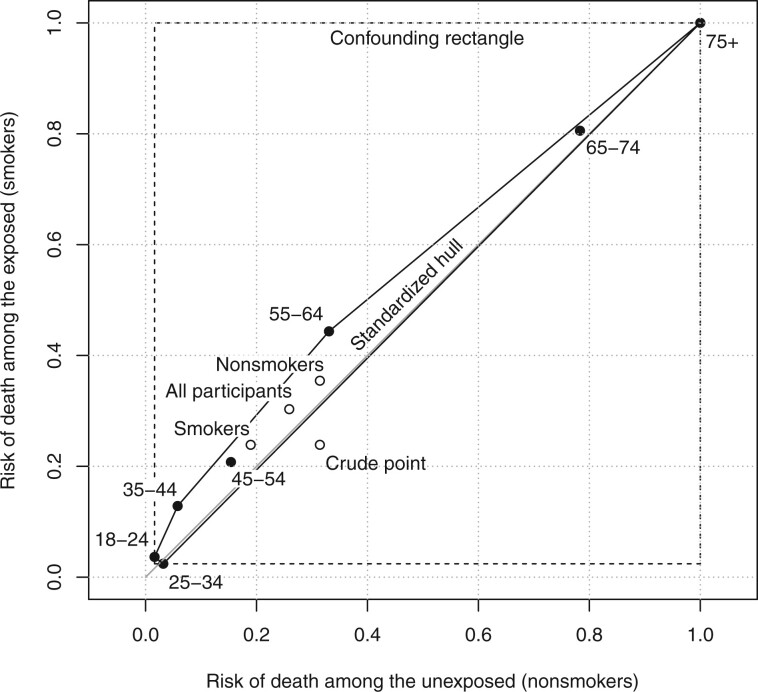
The standardized hull (solid) and the confounding rectangle (dashed) for all seven age strata in Appleton *et al*.^4^ The points standardized to the age distributions of smokers, nonsmokers, and all study participants are inside the standardized hull, but the crude point is outside. As before, the null line is shown in grey

Figure 3 shows the standardized hull for all seven age strata in Appleton *et al.*^4^Supplementary Material, Section S2, available as Supplementary data at *IJE* online shows the corresponding 2×2 tables. The standardized hull contains the points standardized to the age distributions of smokers, nonsmokers, and all study participants. Almost all of the standardized hull is above the null line, so almost all standardized estimates produce a higher risk of death among smokers than nonsmokers. The crude point is outside the standardized hull due to confounding by age. In this case, the standardized risk difference must be between −0.008 and 0.11 but confounding could generate a risk difference as low as −0.98 (at the bottom right corner) or as high as 0.98 (at the top left corner).

## Geometry before regression

The central role of standardization in causal inference can be obscured by an approach that emphasizes conditional measures of association estimated by outcome regression models. The geometric perspective provided by Rothman diagrams gives standardization its proper place as the fulcrum of causal inference, and it takes advantage of intuitions about physical space rather than abstract symbol manipulation. It generalizes to discrete confounders or combinations of confounders with more than two levels, and it helps motivate inverse probability of treatment weighting as a form of standardization.^12^ Because it is based on estimated risks of disease, it also generalizes to longitudinal data with independent right censoring and left truncation (i.e. delayed entry). If there is selection bias,^13^^,^^14^ all of the results discussed here that require stratum-specific association points to be causal hold when stratifying by C is sufficient to control both confounding and selection bias.

## Supplementary Material

dyae139_Supplementary_Data

## Data Availability

The data and R code^15^ needed to replicate all results are available in the Supplementary Material.
