# Supplementary material for: Rothman diagrams: the geometry of confounding and standardization
Source: Int J Epidemiol. 2024 Nov 14;53(6):dyae139. doi: 10.1093/ije/dyae139 (PMC11565235; doi:10.1093/ije/dyae139)
Supplement: dyae139_Supplementary_Data [file dyae139_supplementary_data.zip › ije-2023-10-1294-File006.pdf]

Supplementary Material
Rothman diagrams: the geometry of confounding and standardization

Eben Kenah

S1 Confounding and crude points

When the causal effect of X on D is confounded by C, the crude association point p^*_{crude} must be off the standardized segment connecting p^*_0 and p^*_1 (unless they share an x- or a y-coordinate) In a causal DAG that contains all three variables, there is an open backdoor path from X to D that goes through C [2]. Because of the open path from C to D that does not go through X, at least one of the conditional risks of disease given X differs between strata of C, so the stratum-specific association points p^*_0 and p^*_1 are different. Because of the open path from C to X, the conditional distribution of C given X is different when $X = 0$ and $X = 1$. Therefore, p^*_0 and p^*_1 define a line segment that does not contain p^*_{crude}. If p^*_0 and p^*_1 have the same x-coordinate (i.e., risk among the unexposed is independent of C) or the same y-coordinate (i.e., risk among the exposed is independent of C), then the confounding rectangle and the standardized segment are the same.

When there is no confounding of the causal effect of X and D by C, the crude point must be on the standardized segment. If there is no open path from C to D, then the stratum-specific association points p^*_0 and p^*_1 are the same, so the confounding rectangle and the standardized segment both collapse to that point. If there is no open path from C to X, then the distribution of C is the same for $X = 1$ and $X = 0$, so p^*_{crude} is on the standardized segment. Therefore, we have either $p^*_{\text{crude}} = p^*_0 = p^*_1$ or p^*_{crude} is on the standardized segment connecting p^*_0 and p^*_1.

Due to sampling variation, we may get a p^*_{crude} that is on the standardized segment despite confounding or a p^*_{crude} that is off the standardized segment despite no confounding. This sampling variation disappears as the sample size $n \to \infty$, so the equivalence between confounding and the crude point being off standardized segment holds exactly in the limit of a large sample from an infinite population. In finite samples, this equivalence holds only approximately.

For $k > 2$, sampling variation can produce a crude point outside the standardized hull when there is no confounding. This disappears in the large-sample limit as $n \to \infty$, so a crude point outside the standardized hull implies confounding. In finite samples, this implication holds only approximately.

S2 Age-stratified tables

The following is adapted from Table 2 in Appleton, French, and Vanderpump [1].

Participants aged 18-24 years	Dead	Alive	Total
Smoker	2	53	55
Nonsmoker	1	61	62
Total	3	114	117

Participants aged 25-34 years	Dead	Alive	Total
Smoker	3	121	124
Nonsmoker	5	152	157
Total	8	273	281

Participants aged 35-44 years	Dead	Alive	Total
Smoker	14	95	109
Nonsmoker	7	114	121
Total	21	209	230

Participants aged 45-54 years	Dead	Alive	Total
Smoker	27	103	130
Nonsmoker	12	66	78
Total	39	169	208

Participants aged 55-64 years	Dead	Alive	Total
Smoker	51	64	115
Nonsmoker	40	81	121
Total	91	145	236

Participants aged 65-74 years	Dead	Alive	Total
Smoker	29	7	36
Nonsmoker	101	28	129
Total	130	35	165

Participants aged $\geq$ 75 years	Dead	Alive	Total
Smoker	13	0	13
Nonsmoker	64	0	64
Total	77	0	77

Table 1: Age-stratified 2×2 tables for smoking and 20-year mortality adapted from [1]. Ages are those at the time of participation in the original survey.

References

[1] Appleton DR, French JM, Vanderpump MPJ. Ignoring a covariate: An example of Simpson's paradox. The American Statistician. 1996;50(4):340–341.

[2] Greenland S, Pearl J, Robins JM. Causal diagrams for epidemiologic research. Epidemiology. 1999;10(1):37–48.
